# SARS-CoV-2 infection elicits a rapid neutralizing antibody response that correlates with disease severity

**DOI:** 10.1038/s41598-021-81862-9

**Published:** 2021-01-28

**Authors:** Benjamin Trinité, Ferran Tarrés-Freixas, Jordi Rodon, Edwards Pradenas, Víctor Urrea, Silvia Marfil, María Luisa Rodríguez de la Concepción, Carlos Ávila-Nieto, Carmen Aguilar-Gurrieri, Ana Barajas, Raquel Ortiz, Roger Paredes, Lourdes Mateu, Alfonso Valencia, Víctor Guallar, Lidia Ruiz, Eulàlia Grau, Marta Massanella, Jordi Puig, Anna Chamorro, Nuria Izquierdo-Useros, Joaquim Segalés, Bonaventura Clotet, Jorge Carrillo, Júlia Vergara-Alert, Julià Blanco

**Affiliations:** 1 Institut de Recerca de La Sida, IrsiCaixa AIDS Research Institute, Germans Trias I Pujol Research Institute (IGTP), Hospital Universitari Germans Trias I Pujol, Can Ruti Campus, Ctra, de Canyet s/n, 2a Planta Maternal, 08916 Badalona, Catalonia Spain; 2 IRTA Centre de Recerca en Sanitat Animal (CReSA, IRTA-UAB), Campus de la UAB, 08193 Bellaterra, Catalonia Spain; 3 Infectious Diseases Department, Fight Against AIDS Foundation (FLS), Germans Trias I Pujol Hospital, Badalona, Catalonia Spain; 4 Barcelona Supercomputing Center, Barcelona, Catalonia Spain; 5 Catalan Institution for Research and Advanced Studies (ICREA), Barcelona, Catalonia Spain; 6 UAB, CReSA (IRTA-UAB), Campus de la UAB, 08193 Bellaterra, Cerdanyola del Vallès, Catalonia Spain; 7 University of Vic–Central University of Catalonia (UVic-UCC), Vic, Catalonia Spain

**Keywords:** Infectious diseases, Immunology, Adaptive immunity

## Abstract

The protective effect of neutralizing antibodies in SARS-CoV-2 infected individuals is not yet well defined. To address this issue, we have analyzed the kinetics of neutralizing antibody responses and their association with disease severity. Between March and May 2020, the prospective KING study enrolled 72 COVID-19+ participants grouped according to disease severity. SARS-CoV-2 infection was diagnosed by serological and virological tests. Plasma neutralizing responses were assessed against replicative virus and pseudoviral particles. Multiple regression and non-parametric tests were used to analyze dependence of parameters. The magnitude of neutralizing titers significantly increased with disease severity. Hospitalized individuals developed higher titers compared to mild-symptomatic and asymptomatic individuals, which together showed titers below the detection limit in 50% of cases. Longitudinal analysis confirmed the strong differences in neutralizing titers between non-hospitalized and hospitalized participants and showed rapid kinetics of appearance of neutralizing antibodies (50% and 80% of maximal activity reached after 11 and 17 days after symptoms onset, respectively) in hospitalized patients. No significant impact of age, gender or treatment on the neutralizing titers was observed in this limited cohort. These data identify a clear association of humoral immunity with disease severity and point to immune mechanisms other than antibodies as relevant players in COVID-19 protection.

## Introduction

In December 2019, a novel severe acute respiratory disease was reported in China^1^. Following the early identification, in January 2020^2^, of the severe acute respiratory syndrome coronavirus 2 (SARS-CoV-2) as the etiologic agent of the Coronavirus disease-19 (COVID-19), the new virus rapidly spread to generate a pandemic with a deep impact in global human health. The virus has caused more than 32,800,000 infections and more than 990,000 deaths (as of September 27th, 2020) despite worldwide restrictions in economic activities and mobility.

This massive impact has prompted an unprecedented research taskforce to define the epidemiological features of SARS-CoV-2 transmission, to identify new antivirals and to develop new vaccines able to generate protective immunity against the virus^3,4^. To guide vaccine development, the understanding of the interplay between the virus and the immune system as well as the definition of protective mechanisms have also been established as research priorities^5^. The current knowledge indicates that COVID-19 patients elicit a rapid humoral response against the virus, all of them seroconverting 19 days after symptom onset, with heterogeneous kinetics of IgM and IgG subclasses^6^. Elicited antibodies show reactivity against multiple viral proteins including the outer Spike (S) protein, which is the target of neutralizing antibodies. These include mainly, but not exclusively, antibodies blocking the binding of the S protein to the ACE-2 receptor through interaction with different epitopes of the receptor binding domain (RBD)^7–13^. These antibodies, which are elicited in most infected individuals, are able to protect golden Syrian hamsters from acquisition of SARS-CoV-2 infection^12,14^, and are thought to play a relevant role in viral clearance after infection^15^. Consistently, different S protein-based vaccines are able to induce neutralizing responses and mediate protection in different animal models^16^. In contrast, the implication of antibodies in exacerbated inflammatory responses and in antibody-dependent enhancement of infection (ADE) phenomena are among the potential drawbacks of the humoral response in COVID-19 patients^15^.

Most of the knowledge generated on humoral responses against SARS-CoV-2 is based on severe/hospitalized patients. However, epidemiological data indicate that up to 80% of infected individuals undergo mild symptoms^17^. Importantly there is an undetermined number of infected individuals (reaching 40% in some studies) that do not develop symptoms^18^. Given the high percentage of mild and subclinical cases, the analysis of these individuals may be valuable to understand the global kinetics of herd immunity against the virus.

Here, we longitudinally assessed 72 patients from North Barcelona area displaying a wide range of clinical manifestations (from critical to asymptomatic infection) and we have systematically evaluated their ability to generate neutralizing antibodies. Our data show a rapid elicitation of neutralizing antibodies in hospitalized patients reaching 80% maximal levels 17 days after symptoms onset. In contrast, mild-symptomatic and asymptomatic patients developed lower and sometimes undetectable neutralizing antibodies. These data associate humoral immunity with disease severity and point to immune mechanisms other than neutralizing antibodies as relevant players in COVID-19 protection.

## Results

### Description of participants

The KING study recruited 78 individuals suspected from COVID-19 symptoms. As shown in Table 1 (and supplementary Fig. 1), six individuals gave negative results in both serologic and molecular diagnostic tests and were included in the control uninfected group, while 72 individuals were found positive for SARS-CoV-2 infection by either serological or nucleic acid detection tests and were monitored longitudinally, when possible. From positive individuals, 32 (44%) did not require hospital admission, most of them were identified by mild symptoms (25 individuals), while seven individuals with no symptoms were identified in routine serologic tests. The hospitalized participants (n = 40) were classified according to severity (cutoff pO_2_ saturation 94%) and need of intensive care (Supplementary Fig. 1). One third showed non-severe infection, while 22 patients (55% of hospitalized individuals) were severely affected and 5 required intensive care. The main characteristics of enrolled individuals are shown in Table 1. Significant differences were observed in gender and age (p < 0.05) between infected subgroups, with women and young participants being more represented in the non-hospitalized group. The main comorbidities in hospitalized patients were high blood pressure (19 out 40 patients, 47.5%) and respiratory diseases (10 out of 40 patients, 25%), while the main treatments were hydroxychloroquine, corticosteroids and available antivirals other than remdesivir (mainly lopinavir). Most patients received combined treatments that also included anti-IL-6 biologics (mainly tocilizumab) and Interferon-ß (Table 1).

**Table 1:** Description of participants.

	Uninfected n = 6	SARS-CoV-2 infected		*p*-value
		Non-Hospitalized n = 32	Hospitalized n = 40	
Gender. Female, N (%)	3 (50)	19 (59)	13 (33)	**0.066**^**a**^
Age (years), Median [IQR]	50 [43–62]	51 [42–55]	63 [56–70]	** < 0.0001**^**b**^
Days from symptoms Median [IQR]	–	27 ^18^–^30^	28 ^13^–^35^	ns^b^
Hospitalization days Median [IQR]	–	–	22 ^16^–^28^	** < 0.001**^**c**^
**Severity n (%)**				
Asymptomatic	NA	7 (22)	0 (0)	
Mild/asymptomatic	NA	25 (78)	0 (0)	
Hospital Non-severe	NA	0 (0)	13 (33)	
Hospital Severe	NA	0 (0)	22 (55)	
Hospital ICU	NA	0 (0)	5 (13)	
**Treatment, N (%)**				
Corticosteroids	0 (0)	0 (0)	20 (50)	
Tocilizumab or equivalent	0 (0)	0 (0)	11 (28)	
OHCQ or CQ	0 (0)	1 (4)	39 (98)	
Type I IFN	0 (0)	0 (0)	8 (20)	
PI	0 (0)	0 (0)	17 (43)	
Exitus, N (%)	0 (0)	0 (0)	4 (10)	

Bold values indicate statistically significant differences.

*NA* Not applicable.

^a^Fisher exact test.

^b^Kruskal–Wallis rank sum test.

^c^Mann Whitney test.

**Figure 1:**
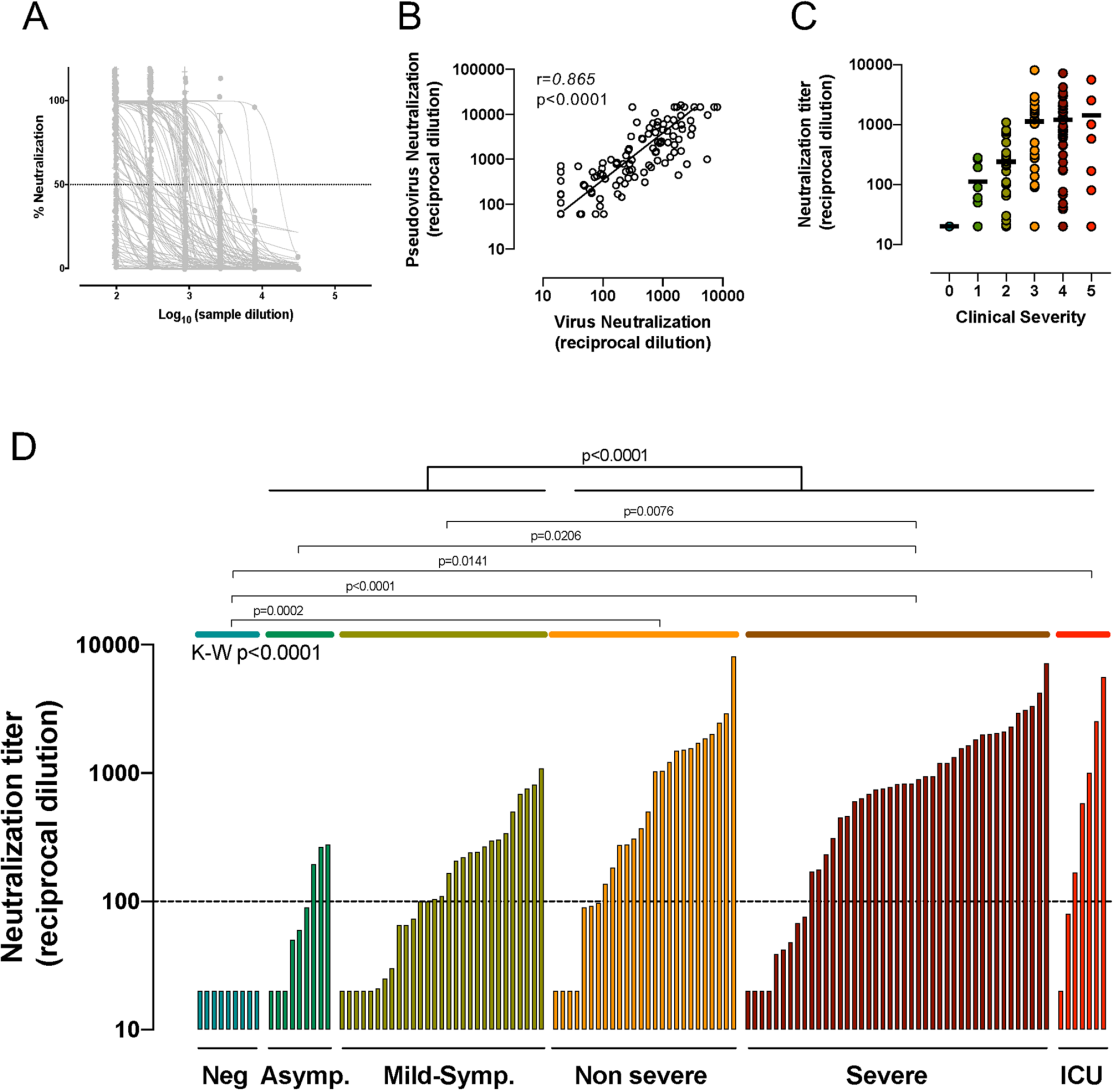
Neutralization activity. (**A**) Dose response of normalized neutralization data for all samples tested against replicative virus in Vero E6 cells (n = 130). (**B**) Correlation between IC_50_ values of plasma samples in replicative virus and pseudovirus neutralization assays (n = 122). Line indicates linear regression for illustrative purposes. Correlation coefficient and p-value (Spearman correlation test) are shown. (**C**) Analysis of the impact of disease severity on neutralization titers (replicative virus assay) for the whole sample set. Individual values, mean values (solid lines) are shown for each group (0 = seronegative, 1 = asymptomatic, 2 = mid-symptomatic, 3 = hospitalized non severe, 4 = severe, 5 = ICU). (**D**) Calculated IC_50_ (reciprocal dilution) in the replicative virus assay for all plasma samples tested grouped by SARS-CoV-2 positivity and clinical grade of symptoms. Comparison between groups was performed by Kruskal–Wallis test (p-value indicated in the Figure) with Dunn’s correction for multiple comparisons (indicated in intergroup comparisons). Top p-value indicates the comparison of the whole hospitalized and outpatient groups.

### Neutralization assays

A total of 128 plasma samples were assayed for neutralization capacity against the replication of an infectious isolate of SARS-CoV-2 in Vero E6 cells (Fig. 1A)^19^ and neutralization titers were determined.

To confirm that neutralization was directly associated with the blockade of S-protein mediated viral entry, a pseudoviral neutralization assay, that uses HIV-based pseudoviruses bearing the SARS-CoV-2 S or the VSV-G proteins, was also developed (see methods). 122 plasma samples were analyzed for pseudovirus neutralization and IC_50_s were compared with the results obtained with the replicative virus neutralization assay. Figure 1B shows the strong correlation between the neutralization titers calculated using each method (r = 0.865, p = 0.00001, Spearman test). This result confirms that plasma-mediated inhibition of fully replicative virus is primarily associated with the presence of neutralizing anti-S antibodies.

Plasma neutralization titers from all infected participants, showed a wide range of activity with a gradual increase in median neutralization activity following disease severity (Fig. 1C). A detailed analysis showed significant differences among disease severity groups (p < 0.0001, Kruskal–Wallis test), that was driven by differences between seronegative individuals and hospitalized subgroups and by significant differences between asymptomatic or mild-symptomatic subgroups with severe patients (Fig. 1D, Dunn’s multiple comparison test). However, no statistical differences were observed between asymptomatic and mild asymptomatic participants or among hospitalized subgroups. When subgroups were combined in non-hospitalized and hospitalized, the former group showed significant lower levels of neutralizing antibodies compared to individuals requiring hospitalization (p < 0.0001, K–W test). Among plasma from infected individuals, 12% of samples reached titers above 2000, with 3 samples, corresponding to three different hospitalized individuals, above 5000. At the other end, 33% of plasma samples showed neutralization titers below 100, mostly corresponding to individuals with mild/asymptomatic infection and early sampled hospitalized individuals (Fig. 1D). All control uninfected individuals showed undetectable neutralizing activity (< 50, reported as 20, Fig. 1D).

### Kinetics of neutralizing antibodies

**Figure 2:**
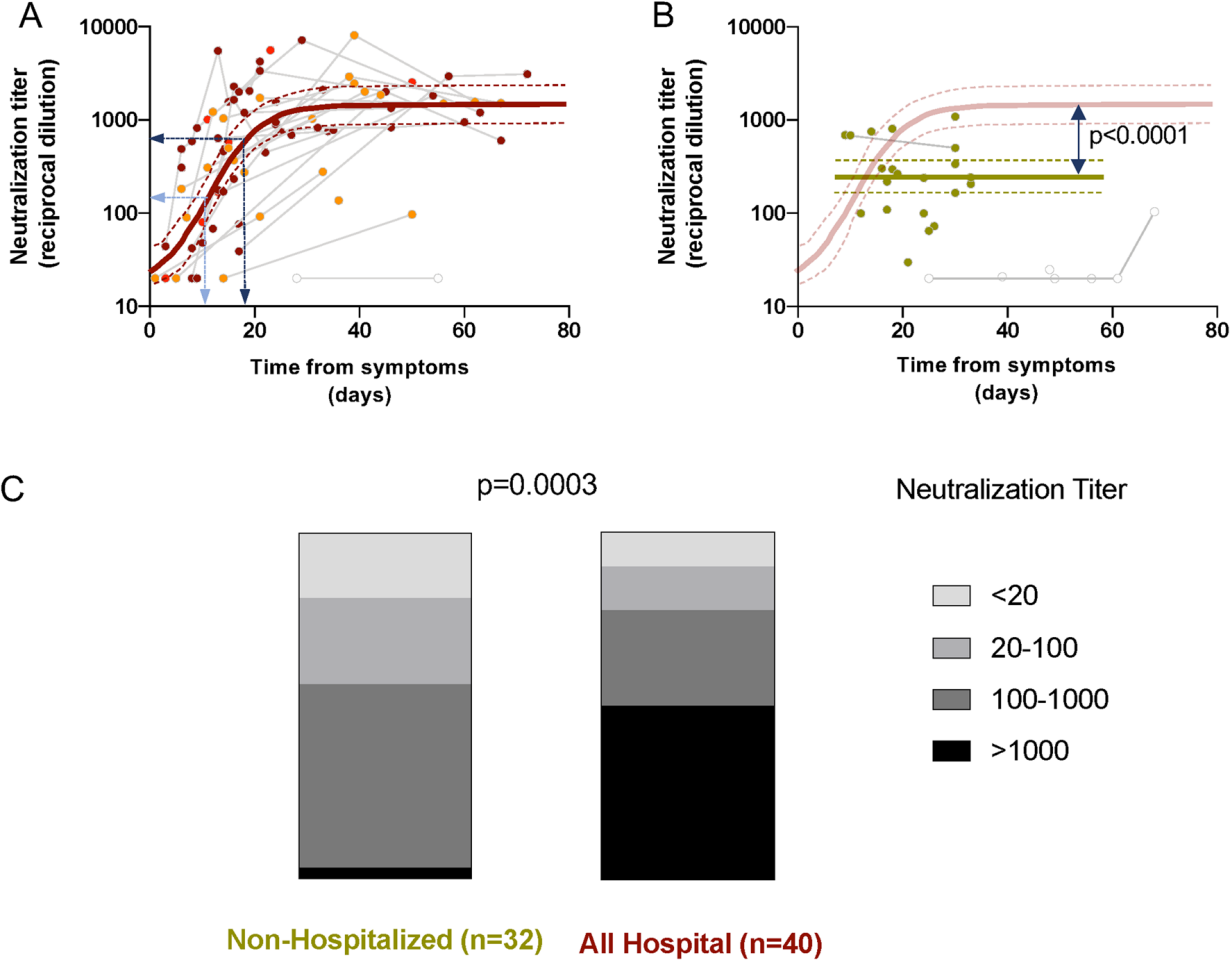
Longitudinal analysis and distribution of neutralization activity. (**A**) Neutralization titers from hospitalized patients were plotted against time from symptoms onset and fitted (solid line). Empty symbols indicate outliers. Light and dark blue arrows indicate the calculated time required to achieve the 50% and the 80% maximal neutralization titer, respectively. Non severe, severe and ICU groups are indicated by orange, maroon and red symbols, respectively. Analysis was performed with all the dataset. (**B**) Neutralization titers from mild-symptomatic individuals were fitted (solid line) after identification of outliers (empty symbols). The comparison of the plateau values for neutralization titers in hospitalized (light maroon line) and mild-symptomatic individuals is shown (Z test). (**C**) Representation of the frequency of undetectable, low, medium and high neutralizing individuals in non-hospitalized and hospitalized (All hospital) patients (p-value of Chi-square test).

Taking advantage of the wide range of sampling times after symptoms onset, we determined the kinetics of emergence of neutralizing antibodies using nonlinear mixed-effects models. Data from hospitalized patients (who had sampling timepoints closer to symptom onset and longer follow-up periods), allowed for proper fitting of data. Kinetics were similar for severe and non-severe individuals, while ICU participants showed a trend towards faster and higher development of neutralizing activity; however, differences were not statistically significant. Fitting all pooled data showed that half maximal neutralization activity was achieved at day 10.7 (confidence interval, CI 8.3–12.9), while 17.3 days (CI 14–21.1) were required to develop the 80% maximal response, which achieved 3.12 logs (CI 2.9–3.3), i.e. 1584 (CI: 794–1995) reciprocal dilution (Fig. 2A). Interestingly, one individual from the hospitalized group failed to generate detectable neutralizing activity even after 55 days of symptoms. This individual was not included in this analysis.

Data from mild-symptomatic individuals could not be analyzed in the same way owing to different temporal distributions of data (as a consequence of difficulties in obtaining samples short term after infection) and low level of neutralizing titers observed in some individuals with late sampling. Therefore, after discarding the late samples, we analyzed the mean neutralization level overtime yielding a value of 2.4 logs (CI 2.2–2.6), i.e. 234 (CI 158–354) reciprocal dilution (Fig. 2B). The difference of this value with the plateau of neutralizing activity of hospitalized individuals was highly significant (p < 10^–7^ by Z-test, and p < 10^–4^ by Wilcoxon test as described in methods; Fig. 2B), and reflects the different distribution (p = 0.0003, Chi-square test) of individuals with undetectable (< 20), low (20–100), medium (100–1000) or high (> 1000) neutralization titers in the non-hospitalized and the hospitalized groups (Fig. 2C). Of note, almost 50% of outpatient (asymptomatic and mild-symptomatic) participants showed low neutralization titers (< 100).

### Association of neutralizing antibodies with age and gender

**Figure 3:**
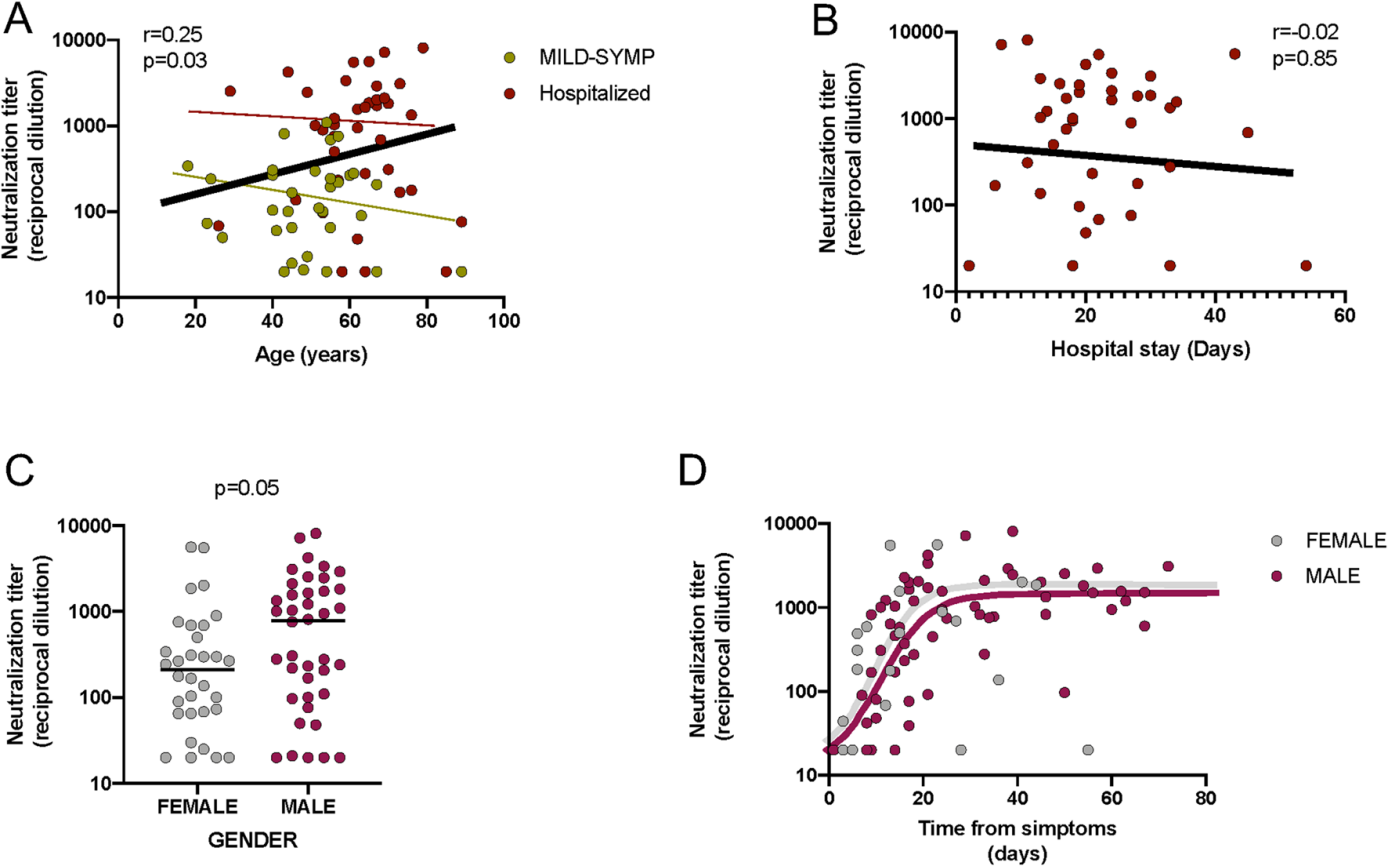
Factors associated with neutralizing responses. (**A**) Correlation between maximal individual neutralizing titers and age. P-value for Spearman’s test correlation of all data is shown (solid line), red and green dotted lines indicate correlations for hospitalized and mild-symptomatic individuals, respectively. *P*-value for Spearman’s test correlation is shown. (**B**) For hospitalized patients, correlation between neutralizing activity and duration of hospital stay. *P*-value for Spearman’s test correlation of data is shown (solid line). (**C**,**D**) Analysis of gender differences in the maximal neutralization titer value of COVID-19 participants (n = 73, **C**) and in hospitalized participants (n = 40, panel D). *P*-values for Mann–Whitney tests is shown.

Since hospitalized and non-hospitalized individuals showed differences in age and gender distribution, we analyzed the impact of these parameters on neutralization titers. A positive correlation was observed between maximal individual neutralization titers and age when all individuals were analyzed (p = 0.03, Spearman test, Fig. 3A). However, significance was lost when each group (hospitalized and non-hospitalized) was analyzed separately (Fig. 3A, dotted lines), suggesting that the main driver of the correlation is the increased age in hospitalized patients. A two-factor regression model, including age and hospitalization status, showed a strong correlation of neutralizing titers with hospitalization (p = 0.0001, Wald test) and a non-significant contribution of age (Table 2). Although we cannot rule out an effect of age due to the limited size of our dataset, these data suggest that severity is the major correlate of neutralizing antibody titer.

**Table 2:** Two-factor regression model to assess the impact of age and hospitalization on neutralizing activity.

	Regression coefficient	Standard error	*p*-value (Wald test)
Age	− 0.004	0.006	0.523
Hospitalization	0.752	0.182	0.0001

For hospitalized patients no correlation was observed between the neutralization capacity and the duration of hospital stay (Fig. 3B). Similarly, unbalanced gender distribution among groups seems to be unrelated to neutralization titer, although barely significant differences were observed when maximal titers of neutralization were compared (Fig. 3C), the kinetics and plateau of female and male participants were similar when a longitudinal analysis was performed (Fig. 3D).

### Impact of treatment on neutralizing titers

**Figure 4:**
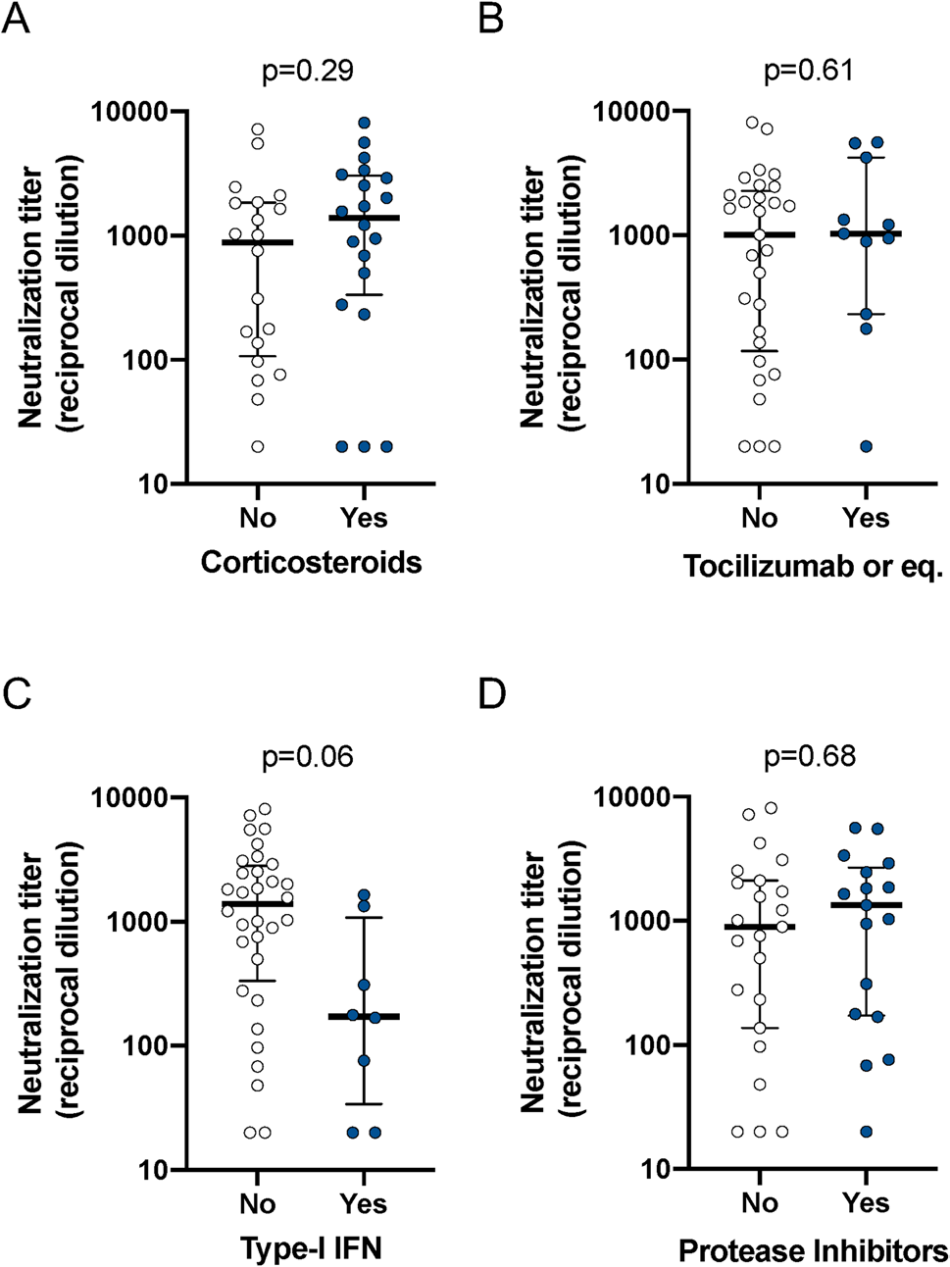
Effect of treatment. Maximal neutralization titers from hospitalized participants (n = 40) were analyzed according to the indicated treatments. Individual values, median and interquartile boxes (25–75) are indicated. *P*-values for Mann–Whitney tests are shown.

We analyzed the potential impact of immunomodulatory or antiviral treatments on neutralizing titers. All participants, but one, were on hydroxychloroquine or chloroquine treatment, hampering the analysis of the effect of this drug. For other drugs, analysis was also perturbed by the different combinations administered. When drugs were analyzed individually, no differences were observed between maximal neutralization titers among participants treated with corticosteroids, tocilizumab (or other anti-IL-6 drugs), type-I IFN (mainly IFN-ß) or protease inhibitors (mainly Lopinavir, Fig. 4). Although type-I IFN seemed to negatively impact neutralization titers, this observation is caused by the high incidence of death (4 out of 8 patients) and the shorter sampling time in the IFN-treated group. We approached the analysis of drug combinations by a more general clustering analysis. However, the large amount of combinations and the limited number of participants prevented the identification of any significant relationships between severity, neutralization titer and treatment regimen (Supplementary Fig. 2).

## Discussion

In this study, we analyzed the development of antibody-mediated neutralizing activity in SARS-CoV-2 infected individuals. We used, either a fully replicative SARS-CoV-2 isolate or a HIV-based pseudovirus exposing the SARS-CoV-2 S protein, similar to other recently reported assays^20–22^. The comparison of both methods yielded a high degree of identity, suggesting that the antiviral activity of plasmas samples is mostly mediated by anti-S protein antibodies (the only SARS-CoV-2 derived protein expressed on the pseudovirus). This comparison also validates the pseudoviral assay as a faster, safer and specific (compared to VSV-G pseudoviruses as control) neutralization screening method.

Our analysis of SARS-CoV-2 infected individuals highlights the association between the development of the neutralizing activity and the clinical course of the infection. First, disease severity appears to be linked to age and gender, with hospitalization rates being higher in both older and male individuals. However, the sub-analyses of hospitalized patients showed no significant differences in neutralization titers according to gender and age. We consider that we do not have enough sample size to be able to correctly assess these questions; therefore, larger studies with longer follow-up will be needed to properly address this issue. Second, hospitalized patients showed a relatively homogeneous development of neutralizing antibodies reaching titers of 3.12 logs. Only ICU cases showed a trend to elicit faster and higher titers, although no clear causality can be established from our data. The global longitudinal analysis showed 50% of response by day 11 and maximal responses (> 80%) attained by day 17 after symptoms onset. These values are similar to those reported for total antibody titers, with 11 and 16 days, respectively^15^, suggesting that the early humoral response already contains neutralizing antibodies. This is consistent with the identification of neutralizing antibodies with a low somatic hypermutation that can probably arise during the first germinal center reactions^23^. No clear effect of treatment on the short-term neutralizing activity was observed as none of the treatments analyzed (tocilizumab, corticosteroids, type-I IFN or protease inhibitors) were associated with higher or lower magnitude of neutralizing responses. This fact contrasts with reported impact of corticosteroid treatment in long-lasting immunity against SARS-CoV^24^; however, the lack of long-term follow up in our samples impedes a direct comparison. Therefore, we cannot rule out a long-term impact, since immunomodulatory interventions might affect the inflammatory balance and the activation and migration of immune cells to secondary lymphoid organs. Again, the reduced sample size and the large number of treatment combinations limited our ability to assess this issue, larger cohorts with longer follow-up are required.

Importantly, our data show that mild-symptomatic participants exhibited significant lower titers of neutralizing antibodies either analyzed longitudinally or by comparing maximal individual values. Consistently, a relevant fraction, roughly 50%, of mild-symptomatic/asymptomatic patients showed neutralization titers below 100, and among them, a significant fraction of individuals with undetectable activity were also identified. This fact has been also observed by others^23,25–27^; and despite that some neutralizing antibodies have been isolated from those individuals^23^, the reasons and the consequences of such a low neutralizing response remain unclear. Exceptionally, we also identified one hospitalized patient with persistent undetectable neutralization titers, despite undergoing severe infection and 33 days hospital stay before recovering.

An obvious risk for patients with low neutralizing capacity is the possibility of reinfection. Although animal models point against this possibility^28^, several cases have been reported in humans^29,30^, and at least one of them was associated with a poor seroconversion after the initial infection^30^. Dangers of low neutralization titer could be also associated with incomplete antibody mediated protection and ADE, a situation of antibody mediated exacerbation of the infection reported for other coronaviruses^31^. However, this is not the case for individuals with low/undetectable neutralizing activity identified in our study, since they have experienced mild-symptomatic or fully asymptomatic infection. Additionally, the absence of correlation between neutralization capacity and length of hospital stay (in the hospitalized group) could suggests that the presence of neutralizing antibodies is not determinant for the resolution of the disease. This is consistent with published data on SARS-CoV-2^32^, but contrasts with a previous study on SARS-CoV patient linking neutralization capacity with shorter illness^33^. Therefore, our data point to a contradictory situation in which neutralization titers do not associate with clinical benefit. In addition, individuals with low antibody responses, far from the doses reported to be protective in animal models^14^, seem to have been protected against severe infection. This apparent contradiction should be explained by further exploration of other immunological mechanisms of viral control. Specifically, innate and/or T-cell mediated responses might play a key role promoting sufficient protection in the absence of a wide and potent B cell mobilization. While few data exist on the protective role of innate immunity against SARS-CoV-2^34^, a relevant role for T-cell responses has been described^35,36^. The hypothesis of a major role of preexisting SARS-CoV-2 cross reactive T cells is of particular interest in this context. These cells could have arisen in a large fraction (roughly 50%) of SARS-CoV-2 unexposed individuals by previous infections with other human coronaviruses causing common cold^37^ and could mediate cross protection as reported in animal models of SARS-CoV and Middle East respiratory syndrome coronavirus infections^38^. Alternatively, the failure to detect neutralizing activity does not rule out the presence of transient albeit low neutralizing responses, which could be sufficient to control early viral replication. Consistent with this hypothesis, low frequencies of RBD-specific B cells have been identified in low neutralizing individuals^23^.

Given the seemingly relevance of asymptomatic or mild-symptomatic infection in the global COVID-19 pandemic^18^, understanding the mechanisms that control viral pathogenesis will be key to assess the herd immunity (antibody-mediated or not) against SARS-CoV-2.

## Materials and methods

### Participants

We designed the KING observational study at the Hospital Universitari Germans Trias i Pujol (Badalona, Spain) aimed to characterize virological and immunological features of SARS-CoV-2 infection. The study was approved by the Hospital Universitari Germans Trias i Pujol Ethics Committee Board (reference PI-20-122). Participants were enrolled after a positive test of SARS-CoV-2 infection (either virological test performed by RT-qPCR analysis of nasopharyngeal swabs in routine clinical screenings or serological test performed by in-house ELISA of plasma samples). All methods were carried out in accordance with the principles of the Declaration of Helsinki. All participants provided written informed consent before inclusion. Some of the individuals recruited in the KING cohort have been included in a sub-analysis of humoral responses recently submitted (Rodriguez de la Concepción et al., submitted).

Severity of symptoms was defined by the following criteria. Asymptomatic infection (severity level 1), mild-symptomatic infection requiring medical visit but no hospitalization (severity level 2), symptomatic non-severe infection requiring hospitalization with pO2 saturation above 94% (severity level 3), severe infection requiring hospitalization and reaching pO2 saturation values below 94% (severity level 4) and very severe infection requiring hospitalization and further intensive care unit (ICU) admission (severity level 5).

### Samples and COVID-19 tests

When available, nasopharyngeal swabs were obtained at time of inclusion in the study and processed by the routine clinical services. Results were categorized as positive or undetectable (considered negative). No quantitative data on viral load was available from these specimens.

Blood was collected by venipuncture in EDTA vacutainer tubes (BD Bioscience). Plasma was obtained by centrifugation of blood at 1200xg for 10 min and stored at – 80 °C until use. The presence of anti-SARS-CoV-2 antibodies in plasma samples was assayed by ELISA (Rodriguez de la Concepción et al., submitted). Briefly, the anti-6xHis antibody HIS.H8 (2 µg/mL in PBS) was coated overnight at 4 °C in MaxiSorp plates (Nunc). Then, plates were blocked using blocking buffer (BB): PBS/1% of bovine serum albumin (BSA, Miltenyi Biotec) for two hours at room temperature. After that, 50 µL of SARS-CoV-2 S2 subunit at 0.9 µg/mL and recombinant RBD at 0.3 µg/mL (both from SinoBiologicals and prepared in BB), were added and incubated overnight at 4 °C. Plasma samples were incubated at 1/100 dilution in BB for one hour at room temperature. The HRP conjugated- (Fab)_2_ Goat anti-human IgG (Fc specific) (1/20,000), Goat anti-human IgM (1/10,000), and Goat anti-human IgA (alpha chain specific) (1/20,000) (Jackson ImmunoResearch) were used as detection antibodies. The specific signal for each sample was calculated after subtracting the background signal obtained for antigen-free wells. Negative cutoffs were defined by COVID-19 negative samples run in parallel.

### Virus neutralization assay

Plasma samples were inactivated (56 °C, 30 min) before mixing at increasing dilutions (ranging from 1/100 to 1/8100) with 60 TCID_50_/mL of the SARS-CoV-2 isolate Cat01 (accession ID EPI_ISL_418268 at GISAID repository: http://gisaid.org), a concentration that achieves a 50% of cytopathic effect as described previously^19^. Uninfected cells and untreated virus-infected cells were used as negative and positive control of infection respectively. In order to detect any plasma-associated cytotoxicity, Vero E6 cells (ATCC CRL-1586) were equally cultured in the presence of increasing plasma dilutions, but in the absence of virus. Cytopathic or cytotoxic effects of the virus or plasma samples were measured at 3 days post infection, using the CellTiter-Glo luminescent cell viability assay (Promega). Luminescence was measured as relative luminescence units (RLU) in a Fluoroskan Ascent FL luminometer (ThermoFisher Scientific).

Dose response neutralization curves were normalized according to positive and negative controls (% Neutralization = (RLUmax – RLUexperimental)/(RLUmax – RLUmin)*100) and fitted to a four-parameter logistic curve with variable slope using Graph Pad Prism software (v8.3.0). All IC_50_ values are expressed as reciprocal dilution.

### Pseudovirus neutralization assay

HIV reporter pseudoviruses expressing SARS-CoV-2 S protein, and Luciferase were generated. pNL4-3.Luc.R-.E- was obtained from the NIH AIDs repository^39^. SARS-CoV-2.SctΔ19 was generated (Geneart) from the full protein sequence of SARS-CoV-2 spike with a deletion of the last 19 amino acids in C-terminal^40^, human-codon optimized and inserted into pcDNA3.4-TOPO.

Expi293F cells were transfected using Expifectamine Reagent (Thermo Fisher Scientific, Waltham, MA, USA) with pNL4-3.Luc.R-.E- and SARS-CoV-2.SctΔ19 at a 24:1 ratio, respectively. Control pseudoviruses were obtained by replacing the S protein expression plasmid by a VSV-G protein expression plasmid as reported previously^41^. Supernatants were harvested 48 h after transfection, filtered at 0.45 µm, frozen and titrated on HEK293T cells overexpressing WT human ACE-2 (Integral Molecular, USA). For neutralization assay, 200 TCID_50_ of pseudovirus supernatant was preincubated with serial dilutions of the heat-inactivated plasma samples (see above) for 1 h at 37 °C and then added onto ACE2 overexpressing HEK293T cells. After 48 h, cells were lysed with Britelite Plus Luciferase reagent (Perkin Elmer, Waltham, MA, USA). Luminescence was measured for 0·2 s with an EnSight Multimode Plate Reader (Perkin Elmer).

Neutralization capacity of the plasma samples was calculated by comparing the experimental RLU calculated from infected cells treated with each plasma to the max RLUs (maximal infectivity calculated from untreated infected cells) and min RLUs (minimal infectivity calculated from uninfected cells), and expressed as percent neutralization: %Neutralization = (RLU_max_–RLU_experimental_)/(RLU_max_–RLU_min_)*100. IC_50_ values were calculated as described above.

### Statistical analysis

Continuous variables were descriptively summarized using medians with 25^th^ and 75^th^ percentiles, and categorical factors were reported using percentages. T-test and chi-square test were used to analyze association of age and gender with the clinical severity of the infection. Association of age with neutralizing titers was analyzed fitting a multivariate linear regression adjusted by clinical severity. We used nonlinear mixed-effects models with an individual based single-level of grouping to model the levels of neutralizing antibodies overtime, estimated since the apparition of symptoms. Models were fitted to a four-parameter logistic function with a constrained lower asymptote set to the limit of detection and three parameters, the inflection point, a scale parameter and the upper asymptote. Individual-specific random effect for upper asymptote was introduced in the model and a first order autocorrelation structure was used to model the within-individuals error variance–covariance structure. In order to analyze differences in antibody concentration between genders and patients with different severity levels, models with covariate-dependent fixed effects were also fitted. Due to the lack of early timepoints in the mid-symptomatic individuals, this group was analyzed separately, estimating the mean level and its standard error of neutralizing antibodies. Comparison of neutralizing antibodies levels between mid-symptomatic and hospitalized groups was assessed in to ways, performing a Z test from estimations and their standard errors (mean level for the former and upper asymptote estimation for the latter) and using Wilcoxon rank sum test to compare antibody levels between mild-symptomatic and hospitalized individuals after 14 days (estimated lower bound to reach the 80% of neutralization level). One individual from the hospitalized group and three from the mild-symptomatic group who failed to generate detectable neutralizing activity were not included in the longitudinal analyses. All analyses were performed with GraphPad Prism 8.4.3 (GraphPad Software, Inc., San Diego, CA) and R version 4.0 (R Foundation for Statistical Computing)^42^. Mixed-effects models was fitted using “nlme” R package.

## Supplementary information

Supplementary figures.
