# Supplementary material for: SARS-CoV-2 infection elicits a rapid neutralizing antibody response that correlates with disease severity
Source: Sci Rep. 2021 Jan 28;11:2608. doi: 10.1038/s41598-021-81862-9 (PMC7843981; doi:10.1038/s41598-021-81862-9)
Supplement: Supplementary file 1 — Supplementary figures. [file 41598_2021_81862_MOESM1_ESM.pdf]

SARS-CoV-2 infection elicits a rapid neutralizing antibody response that correlates with disease severity

Benjamin Trinité[1], Ferran Tarrés-Freixas[1], Jordi Rodon[2], Edwards Pradenas[1], Victor Urrea[1], Silvia Marfil[1], Maria Luisa Rodriguez de la Concepción[1], Carlos Ávila-Nieto[1], Carmen Aguilar-Gurrieri[1], Ana Barajas[1], Raquel Ortiz[1], Roger Paredes[1,3], Lourdes Mateu[3], Alfonso Valencia[4], Victor Guallar[4,5], Lidia Ruiz[1], Eulàlia Grau[1], Marta Massanella[1], Jordi Puig[3], Anna Chamorro[3], Nuria Izquierdo-Useros[1], Joaquim Segalés[2,6], Bonaventura Clotet[1,3,7], Jorge Carrillo[1], Júlia Vergara-Alert[2], Julià Blanco[1,7,*]

[1]IrsiCaixa AIDS Research Institute, Germans Trias i Pujol Research Institute (IGTP), Can Ruti Campus, 08916, Badalona, Catalonia, Spain

[2] IRTA Centre de Recerca en Sanitat Animal (CReSA, IRTA-UAB), Campus de la UAB, 08193 Bellaterra, Catalonia, Spain

[3] Infectious Diseases Department, Fight against AIDS Foundation (FLS), Germans Trias i Pujol Hospital, Badalona, Catalonia, Spain

[4]Barcelona Supercomputing Center, Barcelona, Catalonia, Spain

[5]Catalan Institution for Research and Advanced Studies (ICREA), Barcelona, Catalonia, Spain

[6]UAB, CReSA (IRTA-UAB),Campus de la UAB, 08193 Bellaterra, (Cerdanyola del Vallès), Catalonia, Spain

[7]University of Vic–Central University of Catalonia (UVic-UCC), Vic, Catalonia, Spain

SUPPLEMENTARY MATERIAL

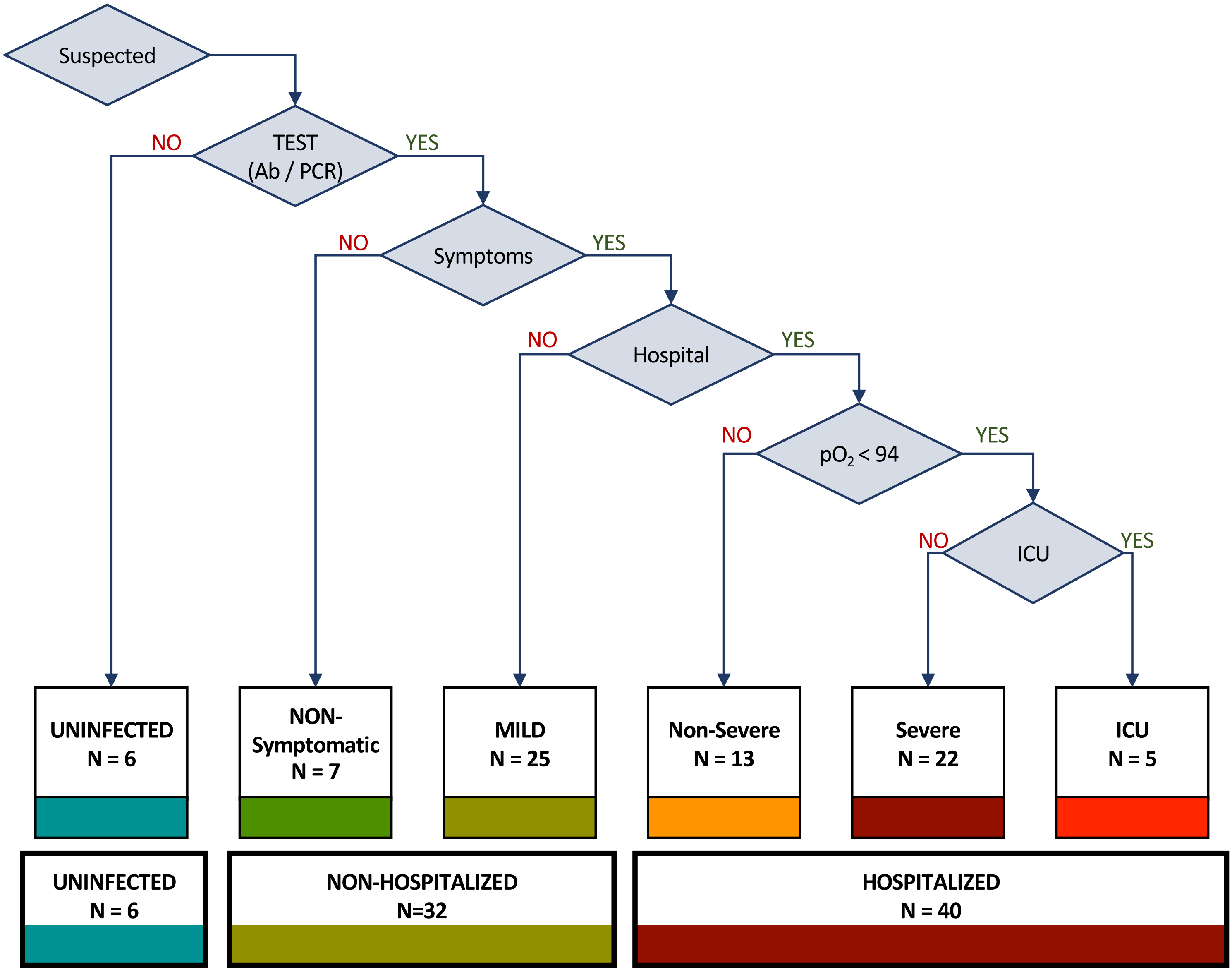

Supplementary Figure 1. Patients classification according to symptoms

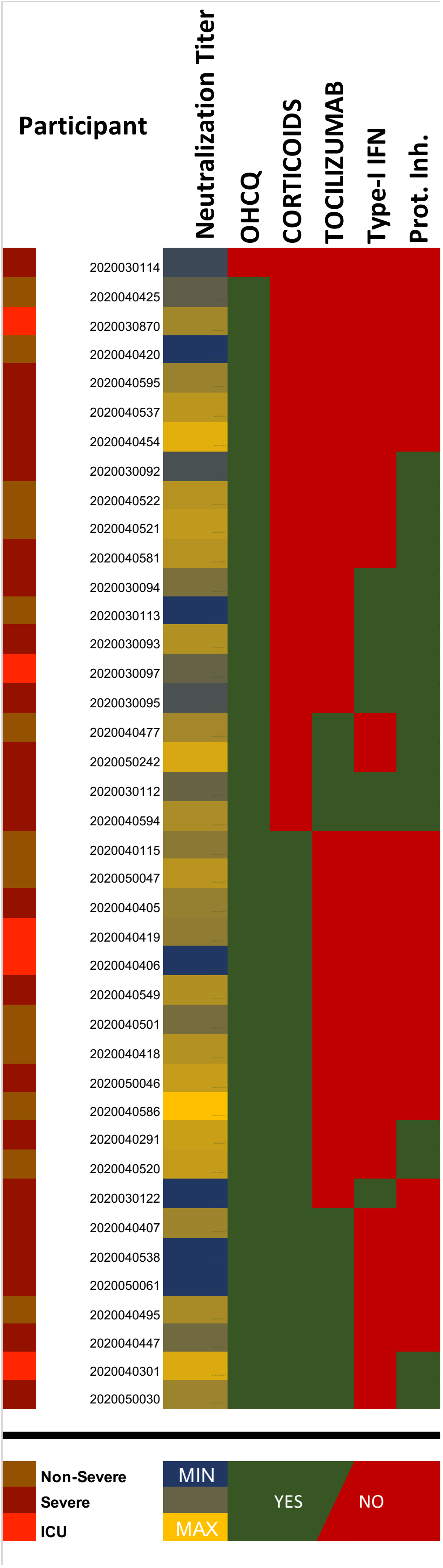

Supplementary Figure 2. Analysis of neutralizing activity according to drug combinations.
